# Effects of FUdR on gene expression in the *C. elegans* bacterial diet OP50

**DOI:** 10.1186/s13104-021-05624-6

**Published:** 2021-05-28

**Authors:** Grace McIntyre, Justin Wright, Hoi Tong Wong, Regina Lamendella, Jason Chan

**Affiliations:** 1 Department of Biology, Marian University, 3200 Cold Spring Rd, Indianapolis, IN 46222 USA; 2 Department of Biology, Juniata College, 1700 Moore St, Huntingdon, PA 16652 USA

**Keywords:** *C. elegans*, *E. coli*, FUdR, Aging, Bacterial transcriptomics

## Abstract

**Objective:**

Many *C. elegans* aging studies use the compound 5-fluro-2ʹ-deoxyuridine (FUdR) to produce a synchronous population of worms. However, the effects of FUdR on the bacterial gene expression of OP50 *E. coli*, the primary laboratory *C. elegans* food source, is not fully understood. This is particularly relevant as studies suggest that intestinal microbes can affect *C. elegans* physiology. Therefore, it is imperative that we understand how exposure to FUdR can affect gene expression changes in OP50 *E. coli.*

**Results:**

An RNAseq dataset comprised of expression patterns of 2900 *E. coli* genes in the strain OP50, which were seeded on either nematode growth media (NGM) plates or on FUdR (50 µM) supplemented NGM plates, was analyzed. Analysis showed differential gene expression in genes involved in general transport, amino acid biosynthesis, transcription, iron transport, and antibiotic resistance. We specifically highlight metabolic enzymes in the l-histidine biosynthesis pathway as differentially expressed between NGM and FUdR exposed OP50. We conclude that OP50 exposed to FUdR results in differential expression of many genes, including those in amino acid biosynthetic pathways.

**Supplementary Information:**

The online version contains supplementary material available at 10.1186/s13104-021-05624-6.

## Introduction

Many *C. elegans* aging studies use the compound 5-fluro-2ʹ-deoxyuridine (FUdR) to produce a synchronous population of worms. FUdR is often added directly to nematode growth media (NGM) agar plates to prevent egg hatching by inhibiting DNA synthesis [1, 2]. However, the effects of FUdR on the bacterial gene expression of OP50 *E. coli*, the primary laboratory *C. elegans* food source, is not fully understood. This is particularly relevant as studies suggest that intestinal microbes can affect host physiology [3–5]. Indeed, bacterial metabolites such as colanic acid extend host lifespan whereas folic acid reduces lifespan [6, 7]. *E. coli* is also known to produce compounds affecting host neuronal function including GABA and lactate which contribute to neuroprotection [8]. While bacterial metabolites are essential to host nutrition and physiology in worms, bacterial load accumulates as the host ages leading to death and a reduced lifespan [9]. Recently, it has also been shown that *E. coli*’*s* metabolic conversion of FUdR to 5-fluorouridine monophosphate (FUMP) can impair *C. elegans* fecundity and highlight the role of bacteria in host health [10–12]. Moreover, FUdR may directly affect *C. elegans* hosts by improving thermal stress response, protein homeostasis, and altering lifespan in some mutant backgrounds [6, 13–15]. Thus, further studies analyzing the gene expression changes in OP50 *E. coli* exposed to FUdR may provide insight into aging and other studies.

## Main text

To assess the role of FUdR on bacterial gene expression changes, we analyzed a dataset of gene expression differences between OP50 *E. coli* seeded on NGM agar plates and on NGM plates supplemented with 50 μM FUdR (NGM + FUdR). This analysis was part of a previous study examining how bacterial gene expression changed in different genetic models of aging, including the longer-lived *daf-2*/insulin-like growth factor receptor and shorter-lived *daf-16*/FOXO transcription factor mutants [16]. The RNAseq data comprised of 2900 *E. coli* genes; Q scores of reads were determined by FastQC and filtered for reads at a 4-base average Q score of 20 or lower using Trimmomatic, as previously described [17]. The raw gene counts (per million) were transformed into a log_2_ scale and cleared of low-quality reads by removing 25% of the probes with the weakest signal. We then identified the top 158 genes with an interquartile range (IQR) > 1.5 (Additional file 1: Table S1). We identified the biological functions of these genes and found genes that are involved in amino acid biosynthesis (32), general transport (28), stress response (13), transcription (12), DNA repair (7), iron transport (6), DNA damage (5), and antibiotic resistance (4). We surmise that some of these genes may be regulated in direct response to FUdR’s effect on DNA.

**Fig. 1:**
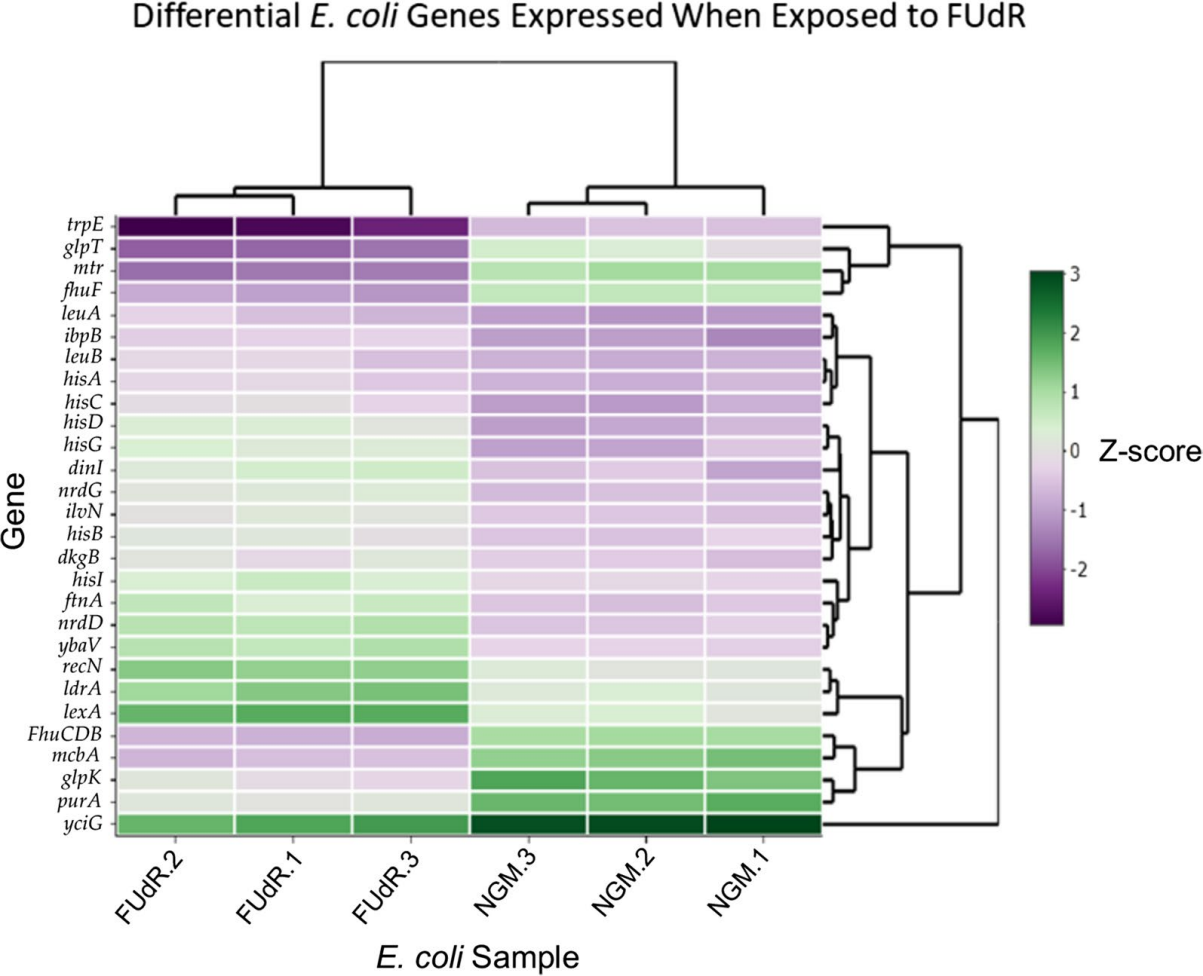
Differentially expressed OP50 *E. coli* genes in response to FUdR. Heatmap demonstrating the top 28 differentially expressed genes in *E. coli* (in triplicate) grown on either NGM only (NGM.1–3) or NGM plates supplemented with 50 µM FudR (FudR.1–3)

**Fig. 2:**
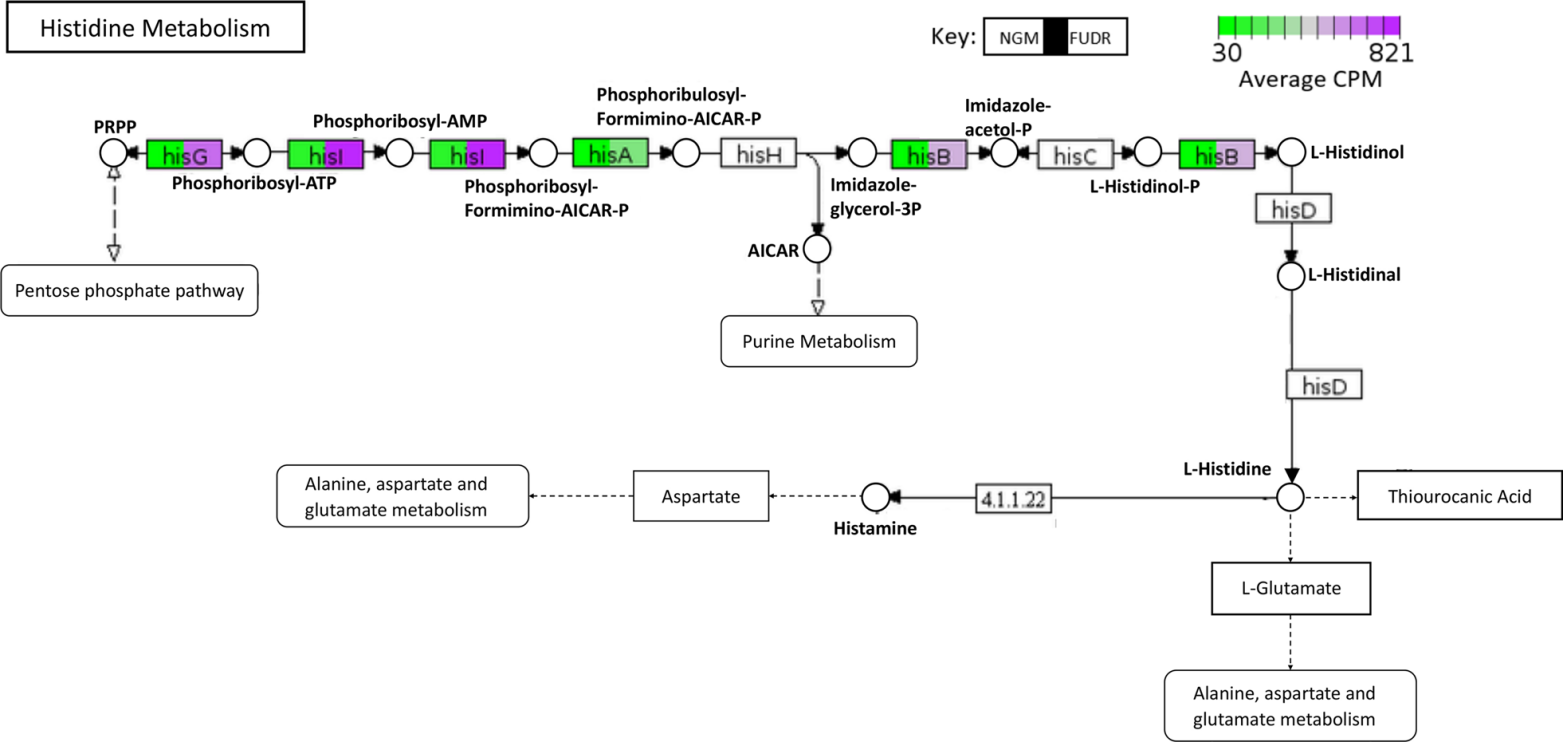
Gene expression differences in the bacterial l-histidine biosynthetic pathway. Pathview plots show differences in the average expression counts of functional enzymes in the l-histidine metabolism pathway between *E. coli* grown on NGM or NGM + FudR plates. The color in the rectangles indicates the average CPM expression of *E. coli* on NGM only (left) compared to *E. coli* exposed to FudR (right). We observed that several enzymes in the l-histidine biosynthesis pathway are upregulated in OP50 *E. coli* when exposed FudR

We further limited our list by examining genes with an IQR > 2.5, which resulted in 28 genes with the greatest differential gene expression (Fig. 1 and Table 1). Hierarchical clustering showed that bacteria from NGM only plates clustered most closely with other NGM only plates, and away from bacteria exposed to FUdR. We found that several of these differentially expressed genes (DEG), including *hisA*,* hisB*,* hisC*,* hisD*,* hisG*, and *hisI*, belong to the l-histidine biosynthesis pathway (Fig. 2). Each of these genes are upregulated in *E. coli* treated with FUdR compared to NGM only. These genes operate under an operon, suggesting that histidine production is increased by FUdR. Histidine can impact cell functions such as energy metabolism and growth, and is necessary in many enzymes as a proton acceptor or donor [18]. Indeed, targeting l-histidine metabolism enzymes may be a therapeutic target to limit survival of *Myobacterium tuberculosis* and other bacteria, suggesting a critical role for histidine in bacterial survival [19, 20]. Previous reports in bacteria also show that histidine starvation can induce metabolomic changes in *E. coli* and that histidine dehydrogenase (*hisD*) is important for bacterial survival against pathogens [18, 21, 22]. Interestingly, histidine is an essential amino acid for worms and can lead to moderate increases in *C. elegans*’ mean lifespan, of *gst-4* and *nhr-57* expression, and resistance to heavy metal toxicity [23, 24]. Given that dietary histidine can have a wide range of benefits for humans, including reduction of inflammation, blood pressure, and metabolic syndrome, our findings may highlight the importance of understanding histidine metabolism in host-bacterial interactions [19].

**Table 1:** Top differentially expressed genes when exposed to 50 µM FUdR

Protein	Gene	LogFC	P-Value	FDR	Function
Amino acid metabolism					
Phosphoribosylformimino 5-aminoimidazole carboxamide ribotide isomerase	*hisA*	2.83	6.29E−23	6.02E−21	l-Histidine biosynthesis
Imidazoleglycerol phosphate dehydrogenase/histidinol phosphate	*hisB*	2.71	3.04E−21	2.38E−19	l-Histidine biosynthesis pathway
Histidinol-phosphate aminotransferase	*hisC*	3.7	9.40E−31	2.21E−28	l-Histidine biosynthesis pathway
Histidinol dehydrogenase	*hidD*	4.19	1.31E−34	3.37E−32	l-Histidine biosynthesis pathway
ATP phosphotibosyltransferase	*hisG*	4.04	9.69E−28	1.56E−25	l-Histidine biosynthesis pathway
Phosphoribosyl atp pyrophosphohydrolase/phosphoribosyl amp cyclohydrolase	*hisI*	2.94	2.00E−24	2.24E−22	l-Histidine biosynthesis pathway
Acetolactate synthase I/III small subunit	*ilvN*	2.93	7.50E−30	1.29E−27	Amino acid biosynthesis, pyruvate fermentation to isobutanol, l-valine biosynthesis
2-Isopropylmalate synthase	*leuA*	3.26	1.22E−23	1.26E−21	3-methylbutanol biosynthesis Pathway, l-leucine biosynthesis
3-Isopropylmalate dehydrogenase	*leuB*	2.91	6.79E−21	5.01E−19	3-methylbutanol biosynthesis pathway, l-leucine biosynthesis
Tryptophan specific transport protein	*mtr*	− 4.57	2.69E−48	1.74E−45	Aromatic amino acid transmembrane transporter activity
Anthranilate synthase component 1	*trpE*	− 2.87	2.81E−24	3.02E−22	Amino acid biosynthesis, l-tryptophan biosynthesis
DNA replication, binding, and repair					
Bacteriocin microcin b17	*mcbA*	− 3.54	1.08E−39	3.10E−37	DNA replication inhibitor, Antibiotic
Repressor lexA	*lexA*	4.37	2.24E−38	1.54E−35	DNA damage, DNA repair, DNA replication, transcription, transcription regulation
DNA repair protein recN	*recN*	3.7	4.30E−40	1.39E−37	DNA damage, DNA repair
Competence protein comea	*ybaV*	3.97	4.98E−50	4.29E−47	DNA binding
Ion transport and signaling					
High affinity iron transporter	*FhuCDB*	− 3.03	1.16E−27	1.76E−25	Ion transport
Ferric iron reductase protein	*fhuF*	− 2.56	8.41E−20	5.71E−18	Colonic acid biosynthesis process
Bacteria non-heme ferritin 1	*ftnA*	3.96	7.42E−42	3.83E−39	Iron storage
Ribonucleoside triphosphate reductase	*nrdD*	4.3	6.53E−54	1.69E−50	Reduces thioredoxin, ATP binding, Zinc ion binding, Nucleotide binding
Anaerobic ribonucleoside triphosphate reductase activating protein	*nrdG*	3.37	7.01E−41	3.02E−38	Metal ion binding, catalyzes 5ʹ-deoxy-adenosine
Other					
DNA damage inducible protein 1	*dinI*	3.86	3.28E−30	6.24E−28	Reductive ion assimilation
2,5-diketo-d-gluconate reductase b	*dkgB*	2.64	2.24E−25	2.75E−23	Ascorbate biosynthesis
Glycerol kinase	*glpK*	− 3.27	1.32E−15	5.01E−14	Glycerol degradation 1 pathway
mfs transporter, opa family, glycerol 3 phosphate transporter	*glpT*	− 3.02	1.15E−17	5.28E−16	Glycerol metabolism, Transport
Small heat shock protein, molecular chaperone ibpB	*ibpB*	3.71	6.52E−52	8.41E−49	Stress response
Small toxic polypeptide	*ldrA*	3.58	2.55E−30	5.48E−28	Toxin–antitoxin system
Anaerobic ribonucleoside triphosphate reductase activating protein	*nrdG*	3.37	7.01E−41	3.02E−38	Metal ion binding, catalyzes 5ʹ-deoxy-adenosine
Adenylosuccinate synthase	*purA*	− 2.75	1.30E−27	1.87E−25	Adenosine ribonucleotides de novo biosynthesis
Uncharacterized protein yciG	*yciG*	− 2.81	5.15E−19	3.17E−17	Bacterial type flagellum dependent swarming motility

A table describing the general biological function of the top 28 differentially expressed genes in *E. coli* with statistically significant (*P* < 0.0001) log fold changes

We also found that FUdR downregulated genes affecting the amino acid tryptophan in OP50 *E. coli*, including the metabolic enzyme antthranilate synthase, *trpE* (2.87-fold decrease, *P* = 2.81 × 10^–24^), and the tryptophan specific transport protein gene, *mtr* (4.56-fold decrease, *P* = 5.44 × 10^–46^). It is unknown whether FUdR affects tryptophan levels in this study, but decreased *trpE* expression may be indicative of elevated l-tryptophan. *trpE* acts as an early step of l-tryptophan biosynthesis and is susceptible to feedback regulation by l-tryptophan [25]. Previous work demonstrated that an increased abundance of tryptophan is associated with increased lifespan in *C. elegans* and microbial tryptophan may serve pro-immune functions in other hosts [26, 27]. However, others show that tryptophan can cause toxicity in worms independent of FUdR, and that pathogenic *E. coli* use tryptophan to produce a secreted toxin that kills hosts [11, 28]. Furthermore, knockout of the transporter *mtr* has been shown to reduce tryptophan intake in *E. coli*, but increase internal l-tryptophan production [29]. Thus, our observations support that FUdR increases l-tryptophan production, and may be part of the mechanism that diminishes *C. elegans* health.

Our analysis also revealed that the iron storage ferritin protein gene, *ftnA* is significantly upregulated by FUdR (3.96-fold increase, *P* = 7.42 × 10^–42^). Furthermore, *fhuF* and *fhuCDB*, which are downregulated by FUdR, are components of ferri-siderophore transport of iron in bacterial membranes. Iron homeostasis is critical for bacteria and other organisms, but it is unclear how FUdR affects iron balance [30]. A lack of intestinal and pharyngeal ferritin in *ftn-1 C. elegans* mutants results in reduced longevity [31]. Importantly, it is not known whether gene expression changes in bacteria ultimately alter bacterial tryptophan or iron abundance. Further studies are needed to specifically address the impact of *E. coli*’*s* l-histidine biosynthesis enzymes, *mtr and trpE* expression, *ftnA* expression, and other differentially expressed genes and the effects FUdR on *C. elegans* physiology and lifespan.

Work examining bacterial metabolism in worms has identified that *E. coli* and *Comamonas* can metabolize FUdR and impact host physiology [10, 11]. Garcia-Gonzalez et al. [10] found that bacteria can either metabolize FUdR to produce fluorouridine monophosphate (FUMP) and affect ribonucleotide metabolism, or to be part of DNA metabolism via FdUMP. FUMP can be synthesized by direct conversion of FUdR into 5-fluorouracil (5-FU) by *deoA* (thymidine phosphoarylase) and conversion of 5-FU into FUMP by *upp* (uracil phosphoribosyltransferase). Interestingly, in our analysis, FUdR treated OP50 resulted in increased expression of both *deoA* (twofold*; P* = 0.7.52 × 10^–6^) and *upp* (twofold, *P* = 2.3 × 10^–4^) (Additional file 2: Figure S1). Furthermore, Ke et al. [11] showed that knockout of thymidine kinase (*tdk*), which directly converts FUdR to FdUMP, enhances the detrimental effects of FUdR. We did not find changes in expression of *tdk* or other downstream enzymes leading to thymidine (dTTP) synthesis (Additional file 2: Figure S1), suggesting that FUdR treated *E. coli* may be driven toward FUMP, not FdUMP production.

Bacteria in *C. elegans* have also been shown to metabolize folate to produce 5-methyl-tetrahydrofolate (5-meTHF), which then impacts methionine metabolism and lifespan in worms [32]. l-Serine to glycine interconversion can produce 5,10-methylene-tetrahydrofolate (5,10-meTHF), a precursor of 5-meTHF, through the enzyme *glyA*. We did not observe changes in gene expression in *glyA*, but we did observe increased gene expression of two l-serine metabolism enzymes, *serA* (3.3-fold increase, *P* = 6.73 × 10^–15^) and *serC* (1.85-fold increase, *P* = 6.45 × 10^–4^) but not of *serB*, which directly synthesizes l-serine (Additional file 2: Figure S1). Thus, it is possible that the substrates necessary to produce 5-meTHF are elevated by FUdR. Furthermore, we did not observe changes in gene expression in thymidylate synthase (*thyA),* which uses 5,10-meTHF convert dUMP to dTMP. Further exploration is necessary to determine the effects of FudR alone on tetrahydrofolate biosynthesis.

Finally, to determine how gene responses to FUdR identified in this study compare to bacterial responses to other factors, we compared our gene list to those previously analyzed. First, our previous report identified a list of core genes in *E. coli* of 4-day-old *C. elegans* hosts. When comparing this to the 158 genes identified in this study, very few overlapped (5/158), suggesting that FUdR initiated a stress specific response. Our previous study also identified differentially expressed bacterial genes in *C. elegans* in various conditions, including temperature and host genotype. We compared those DEGs with genes that were differentially expressed in this study and found 23 common genes regulated by temperature (15 °C compared to 20 °C), 9 by *daf-2*/InsR mutations, and 17 by *daf-16*/FOXO mutations overlapped with our 158 genes (Additional file 3: Table S2). There were very few overlapping DEGs, which may be expected given that our study examined change in bacteria in vitro, and those conditions were in vivo. When comparing our dataset to transcriptomics analysis of *E. coli* exposed to volatile organic compounds, some common pathways regulated iron related gene regulation, including *fhuF*, and leucine biosynthesis [33]. Similarly, *E. coli* treated with the antibiotics potentiator bicarbonate led to increased expression of genes regulating tryptophan and iron, including those in the *fhu* operon [34]. Thus, amino acid and iron regulation may be common responses to chemical treatments, including FUdR.

### Conclusion

In conclusion, these results highlight several genes regulated in OP50 *E. coli* in the presence of FudR. As bacterial diets affect *C. elegans* physiology and longevity, a greater understanding of metabolic networks in bacteria would provide insight to host-microbe interactions. Though this study identifies specific changes in *E. coli* biosynthesis genes and pathways in response to FudR, and further testing of metabolite changes and its impact on host physiology are needed.

### Methods

Sample collection, RNA preparation, sequencing, and gene counts (counts per million) were collected as previously described [16]. Briefly, OP50 *Escherichia coli *(*E. coli*), a standard diet use for laboratory *C. elegans*, was seeded on nematode growth media (NGM) or NGM + 50 µM FudR agar plates. Bacteria were harvested from 3 experimental replicates (3 independent plates from 1 experiment) and prepared for transcriptomics analysis. Genes that mapped to *E. coli* were used for downstream analyses, and gene expression was compared in OP50 bacteria seeded NGM and NGM + FudR (50 µM) agar plates.

#### Pathview analysis

Average normalized CPM counts of identified l-Histidine biosynthesis enzymes were selected using Entrez Reference Sequence identification numbers and mapped to the *E. coli* Histidine Metabolism Pathway (EC00340) using the R package pathview on PathviewWeb [35–40]. Histidine metabolism pathway was simplified with focus on the l-Histidine biosynthesis pathway.

#### Heatmap and genetic analysis

Genes with an IQR > 1.5 produced 158 differentially expressed genes (DEG); (Additional file 1: Table S1). Ninety-two of those DEG were classified based on enzyme parent class and molecular function using EcoCyc and Uniprot databases [41, 42]. Gene counts with an IQR > 2.5 produced 28 DEG which had the greatest expression differences in the presence of FudR based on FDR, logFC, and *P*-value; this analysis was conducted in R 4.0.0 using the package edgeR and heatmaply [43–48]. Gene name, biological pathway, and protein function was determined using MetaCyc, EcoCyc, and Uniprot databases. [41, 42, 49].

#### Reagents

*Bacteria Strain*—*Escherichia coli* strain OP50 provided by CGC (NIH Office of Research Infrastructure Programs (P40 OD010440).

## Limitations

Our analysis of FudR on OP50 *E. coli*’*s* gene expression has identified gene expression differences caused by the presence of FudR. However, this analysis has only identified patterns in gene expression differences. To assess the mechanistic role of FudR on these pathways and downstream metabolites, further studies examining enzymatic changes in *E. coli*’*s* metabolism and its effect on host physiology are needed.

In addition to OP50, other *E. coli* strains including HB101 and HT115 are used as a *C. elegans* food source in lab settings. Furthermore, in the wild, *C. elegans* has been known to feed on a wide variety of bacteria including *Pseudomonas, Ochrobactrum, Methylobacterium, Xanthomonas, and Sphingomonas* [50–53]. As FudR is commonly used in combination with alterations in bacterial diets, further studies investigating the role of FudR in other bacterial strains may provide greater insight into the roles of bacterial metabolites on *C. elegans.*

## Supplementary Information

**Additional file 1: Table S1.** The top 158 differentially expressed genes in OP50* E. coli* exposed to FUdR.

**Additional file 2: Figure S1.** Gene expression differences of biosynthetic pathways for FUdR metabolism.* Top*, FUdR can be metabolized into FUMP or FdUMP. Expression of* deoA* and* upp* are increased (red arrows) in FUdR treated* E. coli*, whereas other metabolic enzmes do not show differential gene expression. (top) and 5,10-meTHF (bottom).* Bottom*, Serine can be metabolized to produce 5,10-meTHF. The expression of l-serine biosynthetic enzymes serA and serC increase in FUdR treated* E. coli*, but expression of enzymes directly snythesizing 5,10-meTHF from l-serine are not changed.

**Additional file 3: Table S2.** Common bacterial genes in OP50* E. coli* regulated in response to FUdR and in specific hosts.

## Data Availability

The datasets analyzed during the current study are available from the corresponding author on reasonable request. The datasets used during this study are included in this published article and its additional information files.
