# Supplementary figures and images for: Effects of FUdR on gene expression in the C. elegans bacterial diet OP50
Source: BMC Res Notes. 2021 May 28;14:207. doi: 10.1186/s13104-021-05624-6 (PMC8186096; doi:10.1186/s13104-021-05624-6)

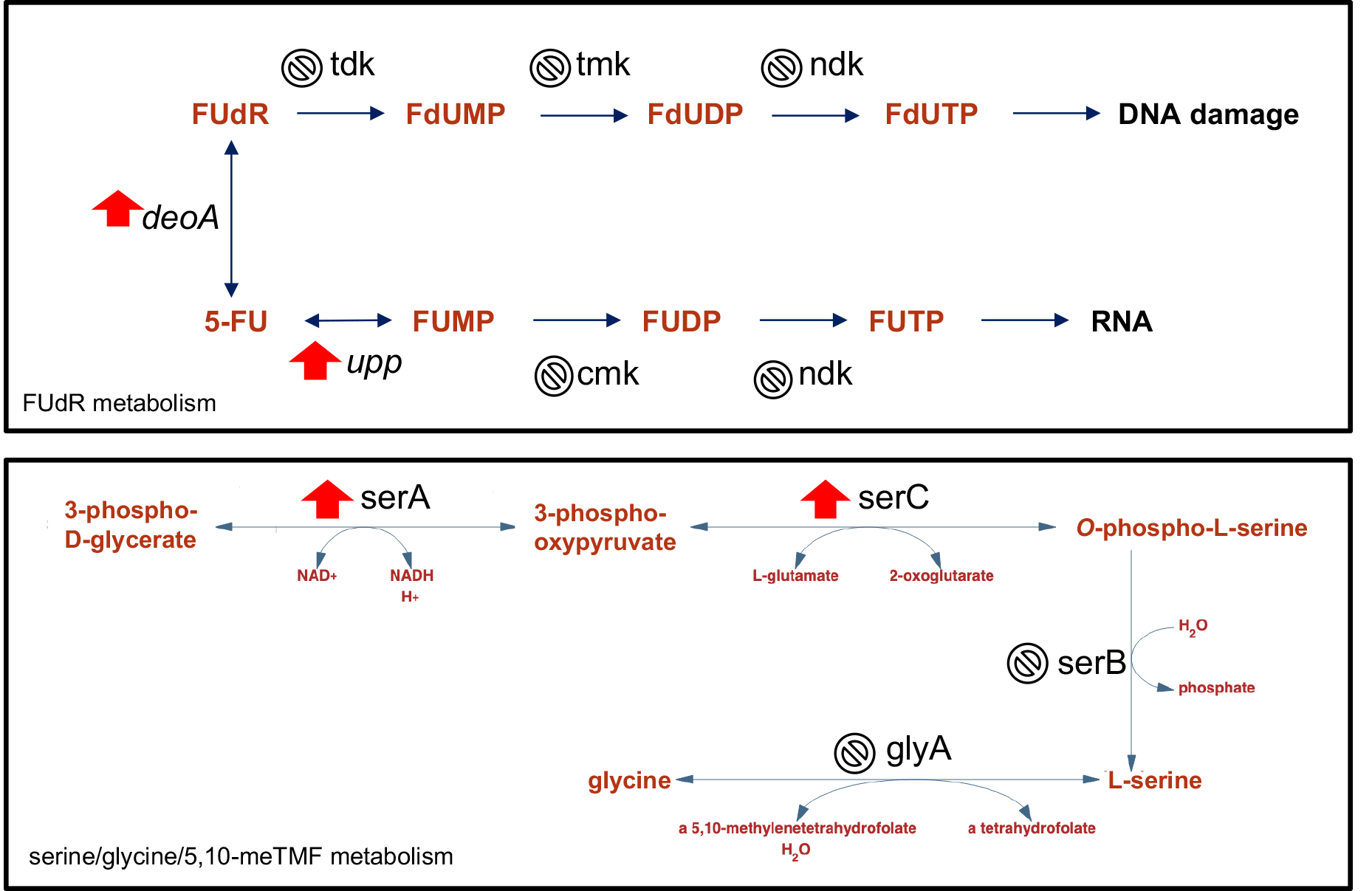

Supplement: Supplementary file 2 — Additional file 2: Figure S1. Gene expression differences of biosynthetic pathways for FUdR metabolism. Top, FUdR can be metabolized into FUMP or FdUMP. Expression of deoA and upp are increased (red arrows) in FUdR treated E. coli, whereas other metabolic enzmes do not show differential gene expression. (top) and 5,10-meTHF (bottom). Bottom, Serine can be metabolized to produce 5,10-meTHF. The expression of l-serine biosynthetic enzymes serA and serC increase in FUdR treated E. coli, but expression of enzymes directly snythesizing 5,10-meTHF from l-serine are not changed. [file 13104_2021_5624_MOESM2_ESM.jpg]
